# Genome-wide identification and expression analysis of the Auxin-Response factor (ARF) gene family in *Medicago sativa* under abiotic stress

**DOI:** 10.1186/s12864-023-09610-z

**Published:** 2023-08-29

**Authors:** Fenqi Chen, Jinqing Zhang, Xue Ha, Huiling Ma

**Affiliations:** College of Pratacultural Science, Gansu Agricultural University, Key Laboratory of Grassland Ecosystem, Ministry of Education, Pratacultural Engineering Laboratory of Gansu Province, Sino-U.S. Center for Grazingland Ecosystem Sustainability, Yingmencun, Anning District, Gansu province Lanzhou, Gansu, 730070 China

**Keywords:** ARF genes, Cultivated alfalfa, Auxin, Abiotic stresses, Expression patterns

## Abstract

**Background:**

Alfalfa (*Medicago sativa*) is the most widely planted legume forage and one of the most economically valuable crops in the world. The periodic changes in its growth and development and abiotic stress determine its yield and economic benefits. Auxin controls many aspects of alfalfa growth by regulating gene expression, including organ differentiation and stress response. Auxin response factors (ARF) are transcription factors that play an essential role in auxin signal transduction and regulate the expression of auxin-responsive genes. However, the function of ARF transcription factors is unclear in autotetraploid-cultivated alfalfa.

**Result:**

A total of 81 *ARF* were identified in the alfalfa genome in this study. Gene Ontology (GO) terms and Kyoto Encyclopedia of Genes and Genomes (KEGG) pathways were analyzed, identifying that *ARF* genes are mainly involved in transcriptional regulation and plant hormone signal transduction pathways. Phylogenetic analysis showed that *MsARF* was divided into four clades: I, II, III, and IV, each containing 52, 13, 7, and 9 genes, respectively. The promoter region of the *MsARF* gene contained stress-related elements, such as ABRE, TC-rich repeats, MBS, LTR. Proteins encoded by 50 *ARF* genes were localized in the nucleus without guide peptides, signal peptides, or transmembrane structures, indicating that most *MsARF* genes are not secreted or transported but only function in the nucleus. Protein structure analysis revealed that the secondary and tertiary structures of the 81 *MsARF* genes varied. Chromosomal localization analysis showed 81 *MsARF* genes were unevenly distributed on 25 chromosomes, with the highest distribution on chromosome 5. Furthermore, 14 segmental duplications and two sets of tandem repeats were identified. Expression analysis indicated that the *MsARF* was differentially expressed in different tissues and under various abiotic stressors. The quantitative reverse transcription polymerase chain reaction (qRT-PCR) analysis showed that the expression profiles of 23 *MsARF* genes were specific to abiotic stresses such as drought, salt, high temperature, and low temperature, as well as tissue-specific and closely related to the duration of stress.

**Conclusion:**

This study identified *MsARF* in the cultivated alfalfa genome based on the autotetraploid level, which GO, KEGG analysis, phylogenetic analysis, sequence characteristics, and expression pattern analysis further confirmed. Together, these findings provide clues for further investigation of *MsARF* functional verification and molecular breeding of alfalfa. This study provides a novel approach to systematically identify and characterize ARF transcription factors in autotetraploid cultivated alfalfa, revealing 23 *MsARF* genes significantly involved in response to various stresses.

**Supplementary Information:**

The online version contains supplementary material available at 10.1186/s12864-023-09610-z.

## Introduction

Auxin is an important plant endogenous hormone that regulates various plant life processes and plays a key role, including tissue differentiation, organogenesis, apical dominance, root formation, tropism, and response to biotic and abiotic stresses [1, 2]. Numerous plants, including *Arabidopsis thaliana*, have a large number of functional genes that are auxin-regulated and play a significant role in growth and development [3–5]. Among these genes, auxin response factor (ARF) family members play a vital role in auxin signaling and regulating the expression of auxin-responsive genes [6, 7]. Most ARFs contain three conserved functional domains: the DNA binding domain (DBD) at the N terminus, the variable intermediate region (MR) acting as the activating domain (AD) or inhibitory domain (RD), and the C-terminal dimerization domain (CTD) [8]. DBD belongs to the b3-like family, which enables ARF to specifically bind to TGTCTC auxin response element (AuxRE) in various auxin response gene promoters, thus transcriptional regulation the expression of these genes widely involved in plant growth and development [9]. Depending on its amino acid composition, MR has fewer sequences and relies on ARF as a transcription activator or repressor. The AD is rich in glutamine, serine, and leucine residues, while the RD is rich in serine, proline, leucine, and glycine residues [8–10]. The CTD is responsible for protein-protein interactions, such as ARF homodimerization or ARF heterodimerization [5]. ARF transcription factors and auxin/indole-3-acetic acid (Aux/IAA) protein dimers can inhibit ARF-regulated transcriptional activity in the presence of low auxin concentrations, but the degree of expression inhibition is only moderate [11]. When the auxin concentrations are high, the 26 S proteasome releases the interacting ARF protein from the inhibitory dimer structure [12, 13].

Furthermore, recent studies have reported relevant information involving interacting proteins and target genes of *ARF*, elucidating the specific mechanisms by which *ARF* regulates plant developmental processes. *Arabidopsis* has 22 *AtARF* functional genes and one pseudogene [8, 14]. The expression of *ARF* is precisely and dynamically regulated in different developmental stages and tissues of *Arabidopsi*s. For example, *AtARF2-4*, *AtARF3*, *AtARF5*, and *AtARF8* are crucial for plant floral organ differentiation and development [15–17]. *AtARF7* and *AtARF19* are essential for auxin-mediated plant development by regulating unique and partially overlapping target gene sets [18]. In addition to regulating plant growth and development, the *ARF* gene family is also involved in plant responses to numerous abiotic stresses.

For example, overexpression of *AtARF3* stimulates the expression of drought-stress-responsive genes in *Arabidopsis*. However, under drought stress, these *ARF*-activated genes maintain very low expression levels in the arf3 mutant [17]. Similarly, the expression levels of most *SlARF* were downregulated under salt stress, while some SlARF genes, such as SlARF1, SlARF4, and *SlARF19*, were significantly upregulated when undergoing salt stress [6]. In lettuce, the expression levels of most *LsARFs* are closely correlated with temperature changes, and *LsARF8a* is involved in regulating the timing of bolting [19]. In earlier studies, authors also found that the accumulation of *LsARF2* and *LsARF5* in flower stems continuously increased with the progress of lettuce bolting and discovered that the tryptophan metabolism pathway was involved in the early bolting of lettuce under high temperatures [20, 21]. In addition, Hu et al. [22] exhibited that *ARF2/26/27/43* actively participates in banana resistance (*Musa acuminata* L.) against cold stress damage. These previous studies have determined that the ARF gene family is essential for regulating plant growth, hormone responses, and responses to abiotic stresses, including drought, salt, and temperature changes, and is a crucial gene family for understanding plant biology.

Alfalfa (*Medicago sativa* L.), known as the “king of forage,” has a high biomass and crude protein content and is rich in digestible nutrients and mineral elements, which dramatically reduces the cost of feed supplements for livestock production [23, 24]. Alfalfa is widely cultivated in North America, Asia, and other continents and is one of the most economically valuable crops in the world [24, 25]. Alfalfa is the fourth most cultivated crop in the United States, after wheat, corn, and soybeans [26]. In China, alfalfa cultivation is distributed through 14 provinces in the northern region of the country, with a locally cultivated alfalfa variety (“Xinjiang Daye”) that has large leaves and an autotetraploid genome (2n = 4x = 32), which is widely cultivated due to its strong resistance [23].

The complete genome data of the autotetraploid variety Xinjiang Daye was released, resulting in a chromosomal-level genome assembly containing 32 high-quality chromosomes instead of the previously assembled 8 chromosomes [26, 27]. The updated assembly of this genome provides an important foundation to facilitate scientific research on this economically important forage crop and improve the stress resistance of alfalfa through genetic engineering. To date, the function of the ARF gene family involved in plant development and abiotic stress has been widely reported in many plants, such as papaya (*Carica papaya*) [9], longan (*Dimocarpus longan* L.) [28], and apple (*Malus domestica*) [29]. Nonetheless, before the report of chen et al. [26], the genome that identified *MsARF* of the cultivated alfalfa is not available. Genomic data and many transcriptome analyses of cultivated alfalfa have been published, providing reliable experimental resources for systematic research on the *MsARF* [30–32]. Based on these data, our study used bioinformatics methods to identify *ARF* in autotetraploid Xinjiang Daye alfalfa at the genome-wide level. We analyzed the phylogeny, gene structure, motif composition, *cis*-regulatory elements, chromosome maps, tissue-specific expression patterns, and differential expression under various abiotic stresses. The results of this study lay the foundation for further investigation of the abiotic stress response and the creation of new germplasm of alfalfa with strong resistance through genetic engineering technology.

## Results

### Identification and functional analysis of *MsARF *family genes

After removing the redundant sequences, a total of 81 MsARF protein sequences were identified in the alfalfa genome and renamed from *MsARF001* to *MsARF081* according to the order of their occurrence in the genome (Table S1). The physicochemical properties of the MsARF proteins, including the gene name, gene ID, amino acid number, molecular weight, isoelectric point, and protein hydrophobicity coefficient, were investigated using ProtParam on the Expasy website (Table S2). Significant differences exist in the number of amino acids, molecular weight, and isoelectric points of the 81 *MsARF* genes. MsARF025 contains 1127 amino acids, making it the gene with the highest number of coding amino acids in the family, with a molecular weight of 126144.77 daltons (Da). MsARF020, with the family’s lowest number of amino acids, encodes 116 amino acids with a molecular weight of 13091.05 Da. The theoretical isoelectric points of these MsARF proteins ranged from 4.32 (MsARF054) to 9.58 (MsARF068). The instability coefficient of the MsARF family ranged from 22.84 (MsARF032) to 98.98 (MsARF039), but the instability index of most genes (66 out of 81) was higher than 40, indicating that they were unstable proteins. The aliphatic amino acid index of 81 MsARF proteins ranged from 48.43 (MsARF067) to 93.76 (MsARF001), all less than 100, indicating that they were hydrophilic proteins. Further subdivided with the help of protein hydrophobicity coefficients, 22 MsARF proteins with hydrophobicity coefficients less than − 0.5 were categorized as hydrophilic, and 59 MsARF proteins were between − 0.5 and 0.5, belonging to the amphotropic protein group.

The GO and KEGG annotation analysis was performed using the Alfalfa Database to determine the functional classification of all *MsARF*. A total of 59 annotated *MsARF* were classified as biological processes, molecular functions, or cellular components (Table S3). Terms like “regulation of nuclear acid template translation,” “regulation of RNA biological process,” and “regulation of translation, DNA template” served as the main annotations for biological processes. In the cellular components, the *MsARF* genes were mainly enriched in terms such as “nucleus”, “internal membrane-bound organelle”, and “membrane-bound organelle”. KEGG enrichment analysis found that 20 *MsARF* genes were enriched in the “plant hormone signal transduction pathway”, and only MsARF039 was enriched in “RNA polymerase”, indicating that *ARF* genes mainly function through the plant hormone signaling pathway (Table S4).

### Phylogenetic analysis of the *MsARF *gene family

**Fig. 1:**
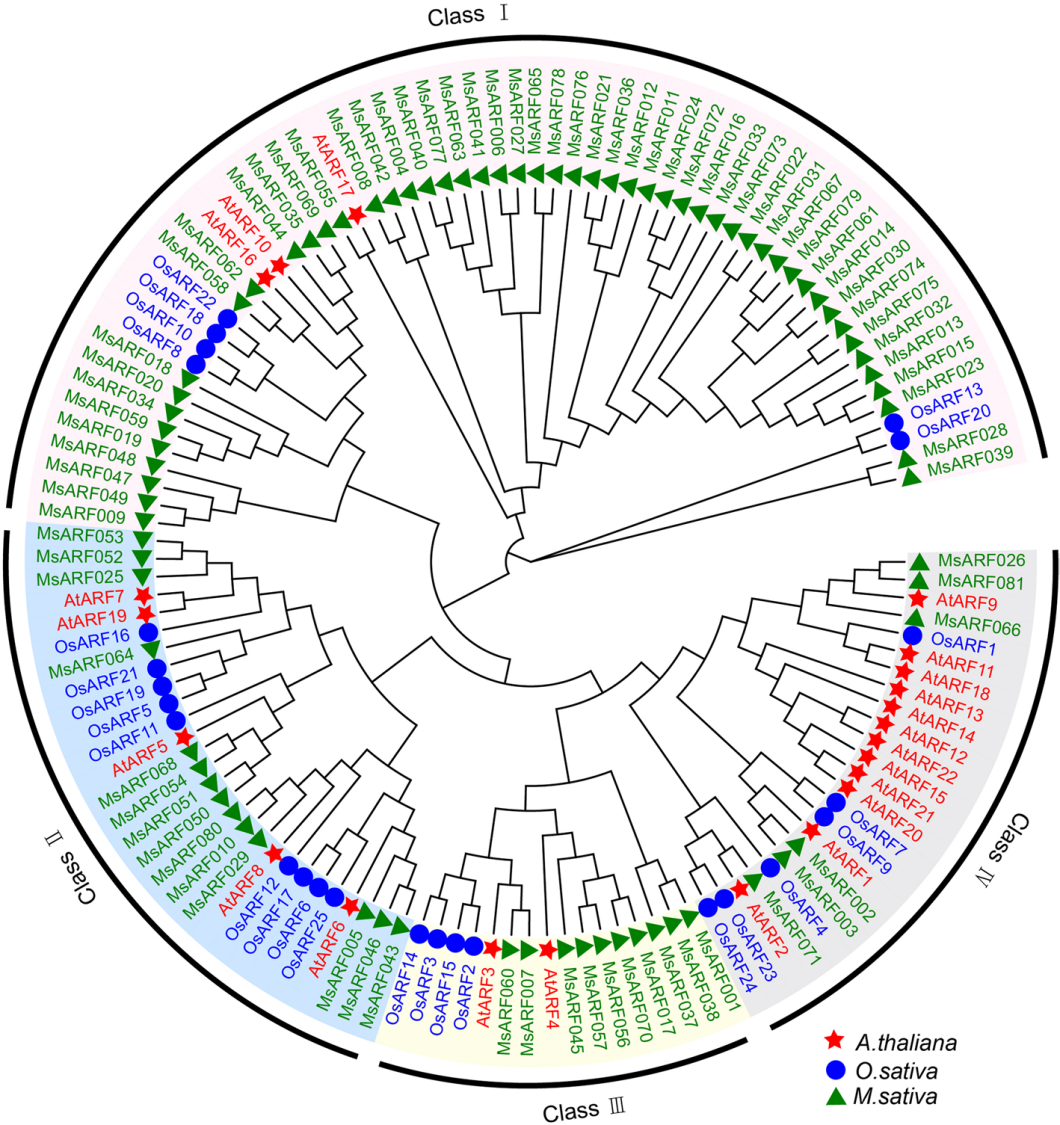
Phylogenetic analysis of ARF protein in *A. thaliana*, *O. sativa*, and *M. sativa*

To study the phylogenetic relationship between MsARF proteins, 25 ARF proteins from rice, 23 from *Arabidopsis*, and 81 from alfalfa identified in this study were selected to further study the phylogenetic relationships of the *MsARF* gene family in alfalfa. The *ARF* gene family was divided into four major clades, of which clade I contained three *Arabidopsis* genes, six rice genes, and 52 alfalfa genes. Clade II contained 5 *Arabidopsis* genes, 9 rice genes, and 13 alfalfa genes. Clade III contains 2 *Arabidopsis* genes, 4 rice genes, and 7 alfalfa genes. Clade IV contains 13 *Arabidopsis* genes, 6 rice genes, and 9 alfalfa genes (Fig. 1).

### Subcellular localization and structural analysis of MsARF protein

**Fig. 2:**
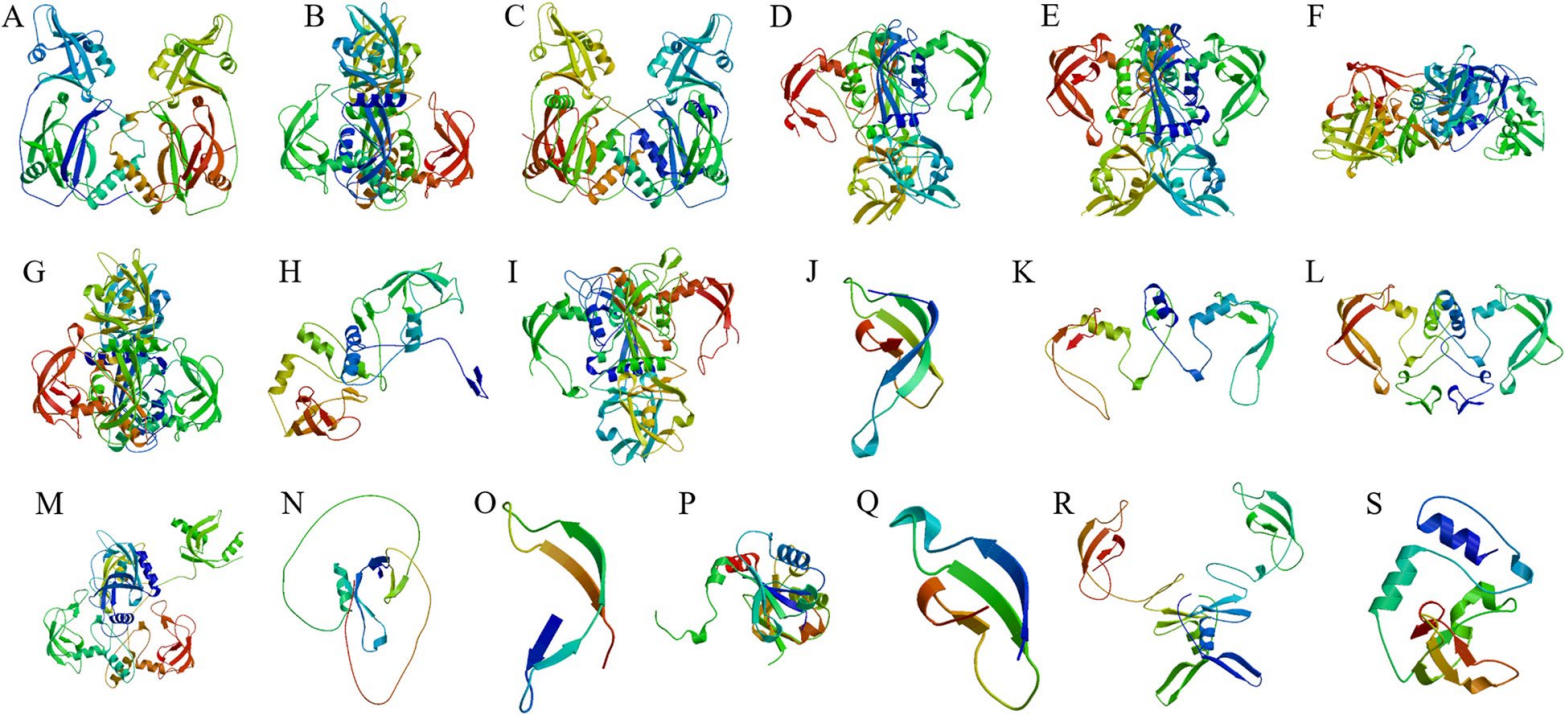
Analysis of MsARF protein tertiary structures. **A** MsARF001; **B** MsARF002, 026, 034, 035, 045, 081; **C** MsARF003, 038, 066, 071; **D** MsARF004, 008,009,012,019,021,025,031,043,047,059,065,078; **E** MsARF005, 010, 029, 046, 050, 052, 053, 064, 080; **F** MsARFS006, 011, 014,015, 022, 023, 027, 032, 033, 036, 037, 040, 056, 057, 060, 061, 063, 067, 070, 074, 075, 076, 077, 079; **G** MsARF007, 017, 055; **H** MsARF013; **I** MsARF016, 024, 041, 042, 044, 048, 058, 062, 069, 073; **J** MsARF018; **K** MsARF020; **L** MsARF028; **M** MsARF030; **N** MsARF039; **O** MsARF049; **P** MsARF051; **Q** MsARF054; **R** MsARF068; **S** MsARF072

Subcellular localization prediction of the *MsARF* was performed, with 22 *MsARF* in the chloroplast, 9 *MsARF* in the cytoplasm, and the remaining 50 *MsARF* in the nucleus (Table S5). The results of the peptide, signal peptide, and transmembrane structure analysis showed that all *MsARF* had no peptides, signal peptides, or transmembrane structures, indicating that the 50 *MsARF* genes located in the nucleus acted as transcription factors, which are not secreted or transported but only function in the nucleus. Analysis of the secondary structures in the MsARF protein found that the largest proportion of the MsARF protein was a random coil, with the composition percentage of random coil > α -helix > extended chain > β -turn angle (Table S5). Simultaneously, the tertiary structure of the MsARF protein was constructed (Fig. 2). Due to the different percentages of secondary structure composition, the tertiary structure also showed significant differences. The specific types of spatial structures in MsARF proteins were relatively few, and most types (46) were obtained through horizontal flipping and rotation, as shown in Fig. 2A C, as well as 2B and 2D, 2E, 2G, and 2I. Another 24 MsARF proteins shared a common spatial framework, as shown in Fig. 2F. In addition, the remaining 11 MsARF proteins all had their own unique spatial framework.

### Conservative motif analysis of the *MsARF* gene

**Fig. 3:**
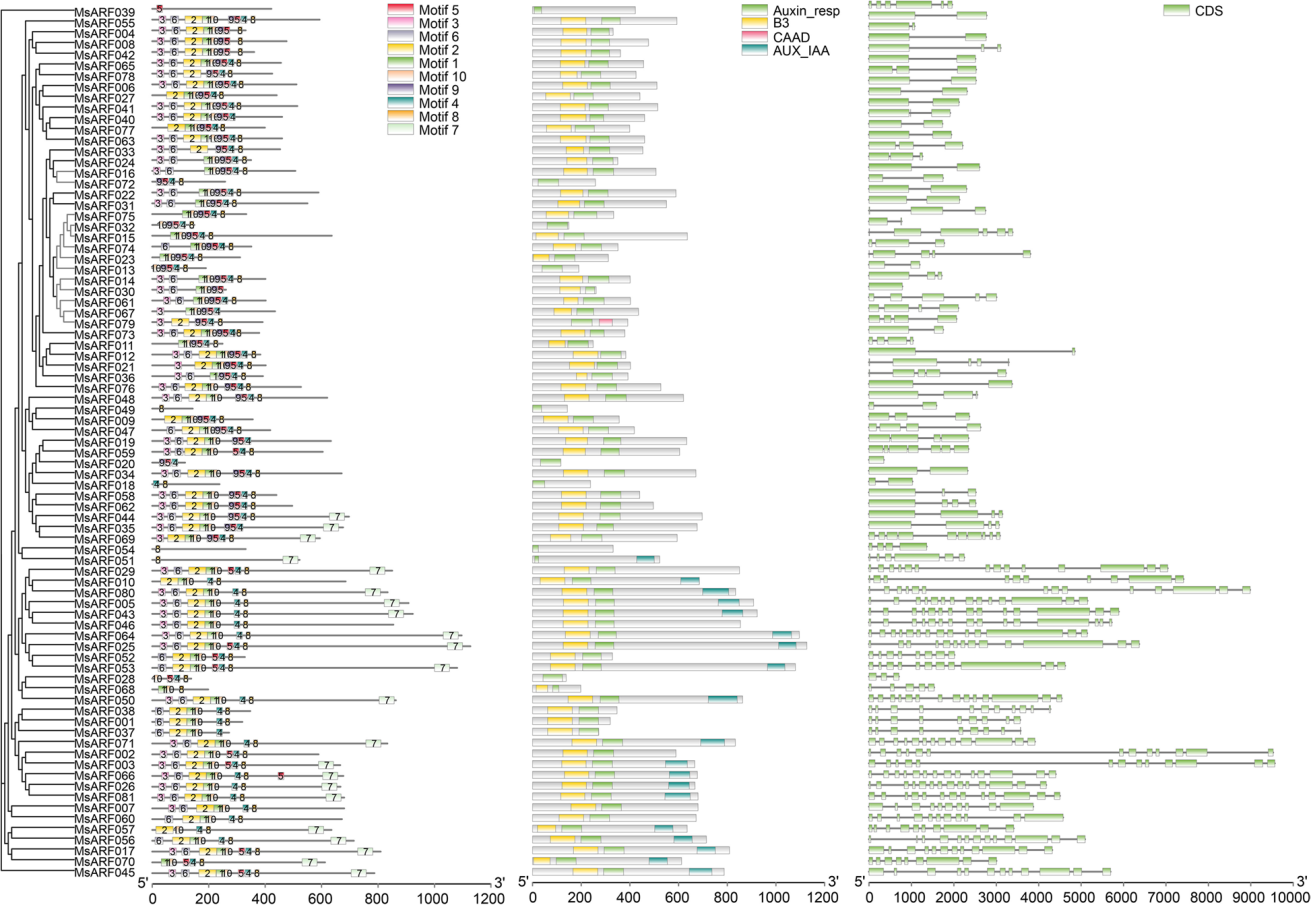
Analysis of conserved moti, domains, and gene structure of MsARF proteins. **A** Gene conservative motif analysis; **B** Protein conservative domain analysis; **C** Gene structure analysis

The *MsARF* domain is the core of ARF transcription factors, which can activate downstream genes by interacting with their promoters. MEME tools were used to explore the distribution and structural diversity of conserved motifs in the MsARF protein and to identify conserved motifs, most of which play important roles in protein-protein interactions and transcriptional activity. A total of 10 conserved motifs were identified and renamed as motifs 1 to 10, with some of these motifs showing similar composition and position in the same subfamily of the *MsARF* (Fig. 3A, Fig. S1). Motif 8 was highly conserved in the *MsARF* gene family and was identified in all 80 *MsARF* except *MsARF039*, indicating that motif 8 is one of the most important motifs in the *MsARF* gene family (Fig. 3). Furthermore, motif 7 was highly conserved in the MsARF subfamilies II, III, and IV.

Analysis of the conserved domains of the MsARF protein sequences revealed that all MsARF sequences contained an auxin response domain (Auxin_resp). Among the 81 MsARF sequences, except for 10 MsARFs (MsARF013/018/028/032/039/049/051/054/072/079), all other sequences contain a typical DBD domain (B3-like). However, phylogenetic analysis revealed that the Aux/IAA binding domain is only distributed in the clade II, III, and IV subfamilies (*n* = 19) (Figs. 1 and 3B). Furthermore, MsARF079 also contains a cyanobacterial aminoacyl-tRNA synthetases appended domain (CAAD). To continue to determine the structural differences among *MsARF*s and reveal gene function, regulation, and evolution, a gene structure analysis of the *MsARF* family was performed. Gene structure analysis showed that the number of CDS in the *MsARF* gene ranged from 1 to 15 (Fig. 3C), and no UTR regions were identified. Among the 81 *MsARF* genes, *MsARF046* has the most CDS (*n* = 15), followed by *MsARF005*, *MsARF026*, *MsARF029*, *MsARF043*, *MsARF050*, *MsARF064*, and *MsARF066* that have 14 CDS, while *MsARF020* and *MsARF030* have only one CDS. Phylogenetic analysis revealed that the *MsARF* genes differ in the number of CDS in different subfamilies.

### Chromosomal localization and homology analysis of *MsARF* gene in alfalfa

**Fig. 4:**
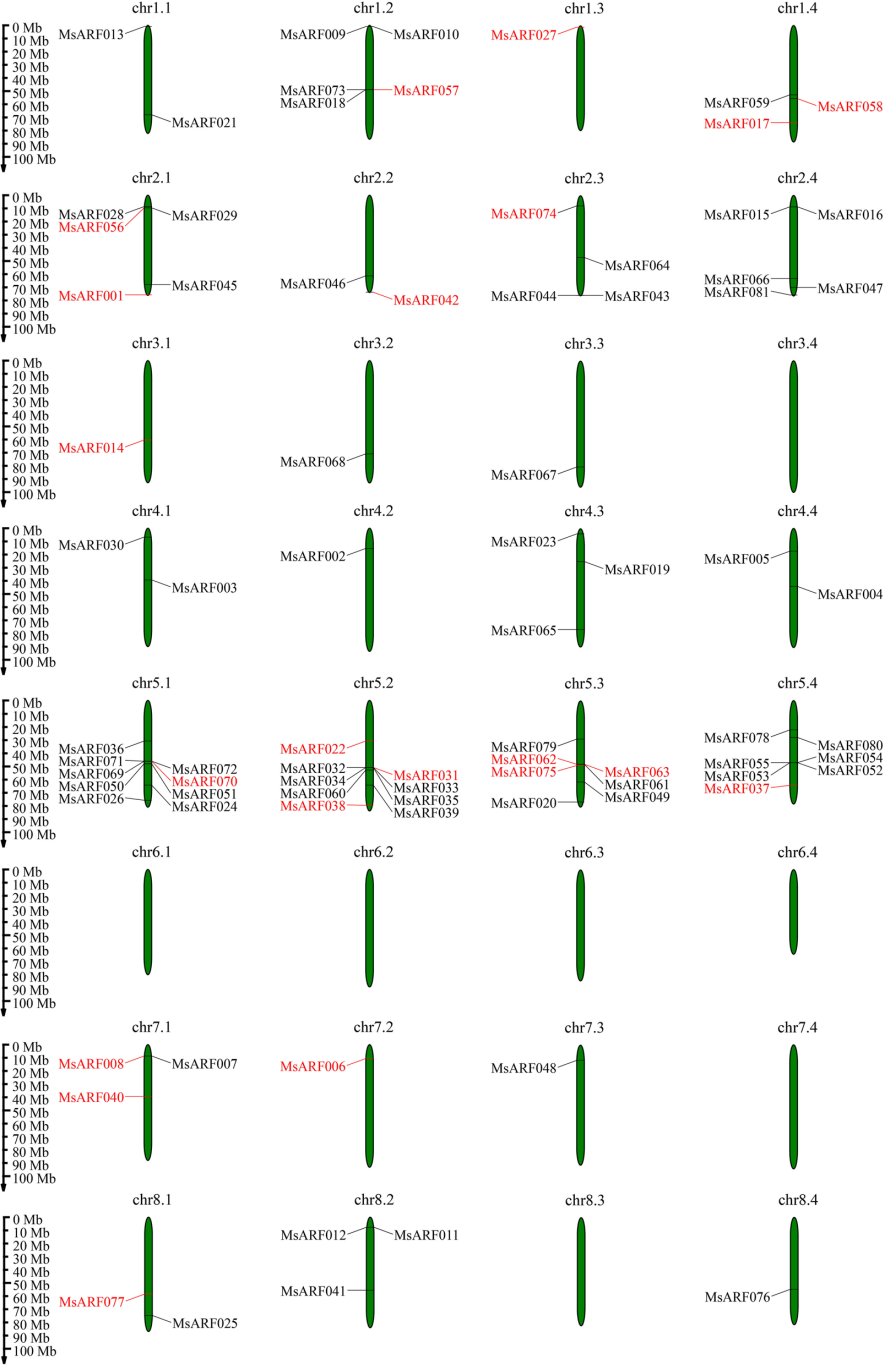
Distribution and location of the *MsARF* gene on alfalfa chromosomes. Chr 1.1 to Chr 8.4 represent the linkage group of “Xinjiang Daye” alfalfa. Each black line indicates the location of the *ARF* gene. The red line represents tandem duplicates of the *ARF* gene

This study identified and mapped the chromosomal positions of all *MsARF* genes to investigate whether there was a collateral homologous gene pair relationship between the *MsARF*s in alfalfa (Fig. 4). The 81 *MsARF* genes were unevenly distributed on 25 chromosomes (Chr) and seven other chromosomes (Chr 3.4, Chr 6.1, Chr 6.2, Chr 6.3, Chr 6.4, Chr 7.4, and Chr 8.3) (Fig. 4). The *MsARF* was distributed the most on Chromosome 5, with Chr 5.1 and Chr 5.2, each containing nine members, and Chr 5.3 and Chr 5.4, each containing seven members. Furthermore, five *MsARF*s were found on Chr 1.2, Chr 2.1, and Chr 2.4, each with four members on Chr 2.3; three *MsARF*s each on Chr 1.4, Chr 1.1, and Chr 8.2; and two *MsARF*s on Chr 1.1, Chr 2.2, Chr 4.1, and Chr 8.1 each. There was only one *MsARF* on the remaining eight chromosomes.

**Fig. 5:**
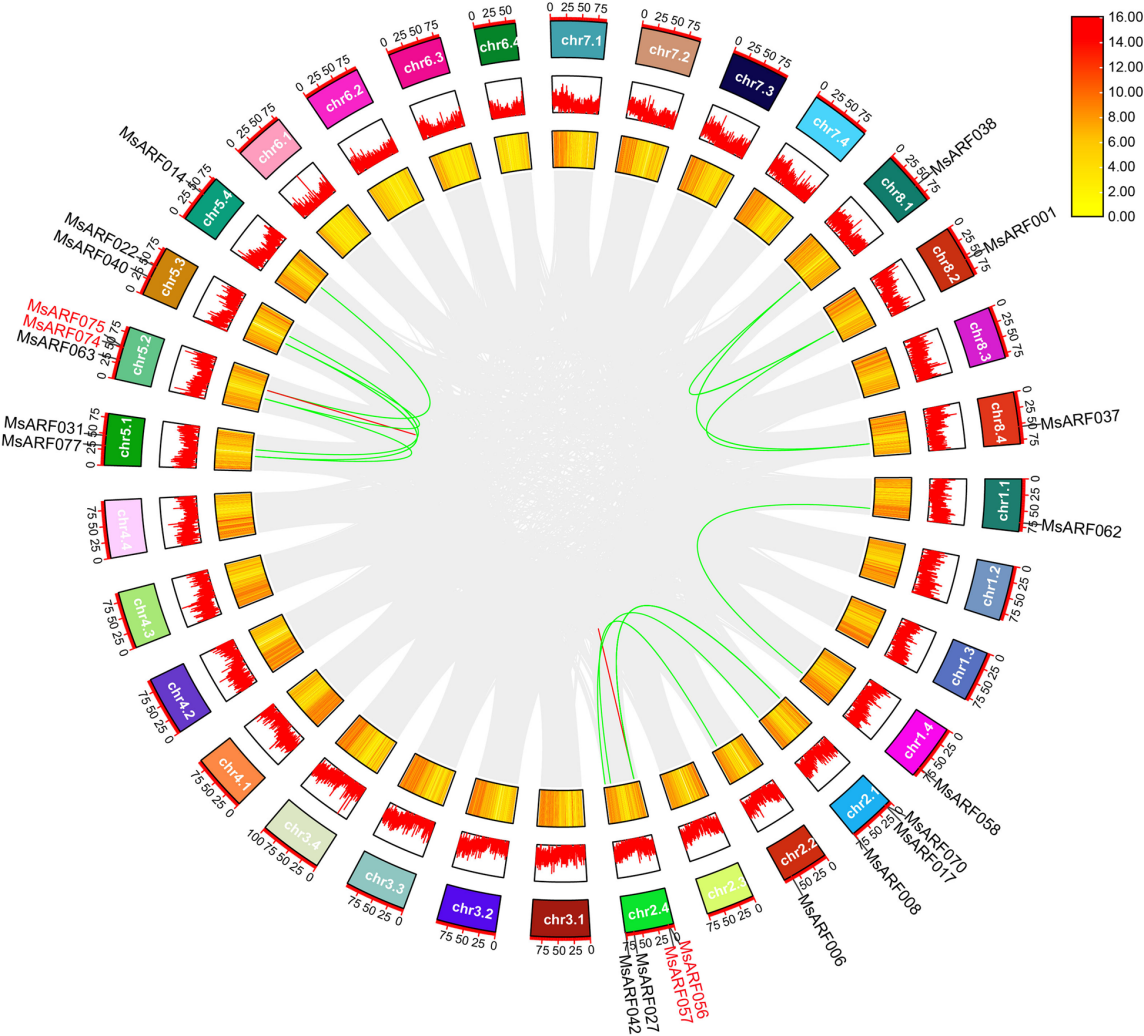
Synteny analysis of the *MsARF* gene. The gray line represents all the synteny blocks in the alfalfa genome. The green and red lines represent gene pairs, and tandem repeat genes in the *ARF* gene, respectively. The yellow heat map and the broken red line map represent the gene density and expression level of the *MsARF* gene, respectively

We used TBtools software to conduct a collinearity analysis to detect gene duplication events in the *ARF* in alfalfa [33]. 14 pairs of segment repeats and two sets of tandem repeats in the *MsARF* were identified (*MsARF056/MsMASS057* and *MsARF074/MsMASS075*) (Fig. 5). The nonsynonymous substitution rate/synonymous substitution rate (Ka/Ks) ratio is usually an important indicator of selection pressure in evolution [34]. The analysis of homologous and homologous *MsARF* gene pairs showed that among the 14 pairs of segment repeats and two sets of tandem repeats of *MsARF* genes, the Ka/Ks ratio of two pairs of segment repeats (*MsARF008/MsMASS042* and *MsARF008/MsMASS042*) was greater than one, indicating a positive selection effect. The Ka/Ks ratios of the remaining 14 pairs of *MsARF* genes were all less than one, indicating they underwent strong purification selection after replication (Table S6).

### Analysis of *Cis*-regulatory elements in the promoter region of the *MsARF *gene family

**Fig. 6:**
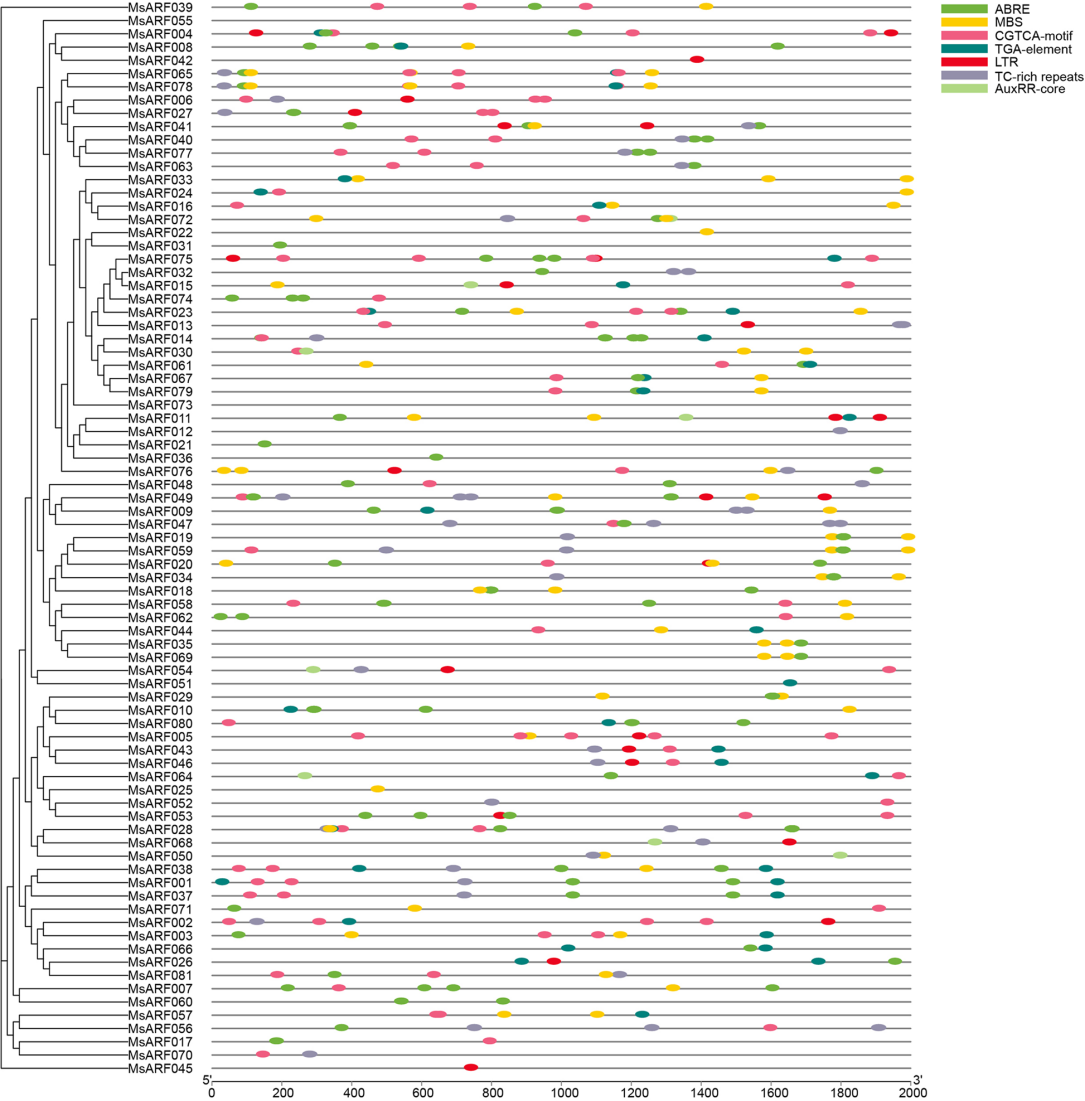
Analysis of *Cis*-regulatory elements in the upstream promoter of the *MsARF* gene

*Cis*-regulatory elements are specific deoxyribonucleic acid (DNA) sequences located upstream of gene coding sequences and regulate the expression of stress response genes by binding to transcription factors. Therefore, we explored the distribution of seven *cis*-regulatory elements in the promoter regions of these altered *MsARF* genes during abiotic stress. We explored the distribution of *cis*-regulatory elements related to hormones and abiotic stress in the *MsARF* gene promoter region (Fig. 6). *cis*-regulatory elements related to hormones include abscisic acid response: ABRE (*n* = 118), Methyl Jasmonate (MeJA) response: CGTCA-motif (*n* = 91), auxin response: TGA-element (n = 38) and AuxRR-core (*n* = 8). *cis*-regulatory elements related to abiotic stress include defense and stress response: TC-rich repeats (*n* = 49), drought induction: MBS (*n* = 66), and low-temperature response: LTR (*n* = 27). In addition, we found that there was only a *cis*-regulatory element in *MsARF012*, *MsARF021*, *MsARF022*, *MsARF025*, *MsARF031*, *MsARF036*, *MsARF042*, *MsARF045* and *MsARF051*.

### Expression pattern analysis of the *MsARF* gene family

**Fig. 7:**
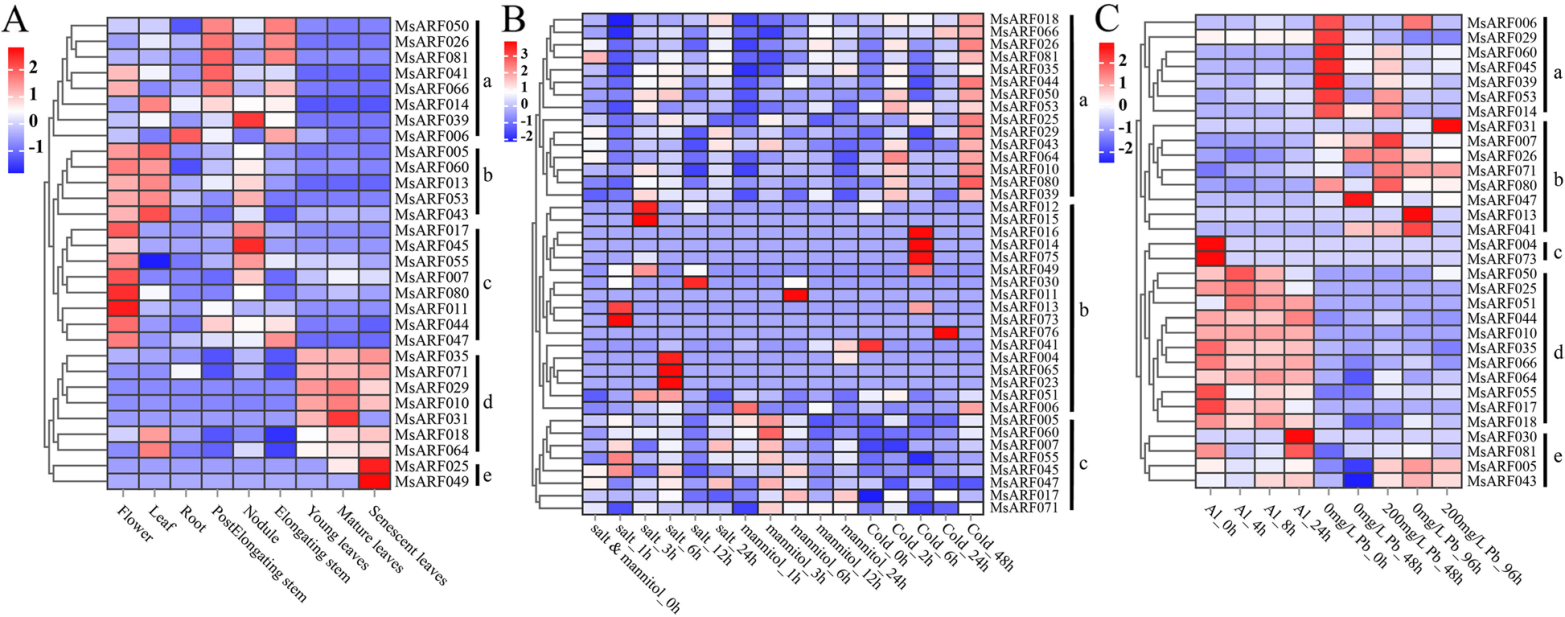
Analysis of the expression pattern of the *MsARF* gene family. **A** Tissue-specific expression pattern analysis; **B** Analysis of expression patterns under salt, drought, and cold stress; **C** Analysis of expression patterns under aluminum (Al) and lead (Pb) stress

Tissue-specific expression is related to the specific function of *ARF* genes in specific tissues. Therefore, 81 *MsARF* genes were compared and identified using ‘blastn’ on the Alfalfa Database, and expression patterns of 45 *MsARFs* were obtained using gene expression profile data (Table S7). TBtools software was used to generate *MsARF* genes (gene expression data was 0 when mapping). The *MsARF* was expressed in eight specific tissues, including flowers, leaves, roots, post-elongating stems, nodules, elongating stems, young leaves, mature leaves, and senescent leaves of alfalfa. Group a genes were highly expressed in flowers, leaves, roots, pre-elongated stems, nodules, and elongated stems. Group b genes were highly expressed in flowers and leaves. Group c genes were highly expressed in flowers, and group d genes were highly expressed in young, mature, and senescent leaves, which may play an important role in leaf development. The two genes in group e, *MsARF025* and *MsARF049*, were highly expressed in senescent leaves and considered putative key regulatory genes for this specific tissue type (Fig. 7A). To identify the expression patterns of *MsARF* family genes in response to abiotic stress, we analyzed the transcriptional expression profiles of *MsARF* family genes under salt stress, mannitol treatment, cold stress, and metal ion stress (aluminum and lead) (Fig. 7B, C). In Fig. 7B, group a genes were mainly upregulated after 48 h of cold stress, participating in alfalfa’s response to cold stress. *MsARF012* and *MsARF015* were mainly overexpressed after 3 h of salt stress. Five *MsARF* genes (*MsARF016*, *MsARF014*, MsARF075, *MsARF049*, and *MsARF013*) were overexpressed after 6 h of cold stress, and *MsARF011* was overexpressed after 6 h of mannitol treatment, suggesting that these genes may play a vital role in alfalfa’s response to abiotic stress.

### Real-time quantitative PCR analysis of the *MsARF* family genes

**Fig. 8:**
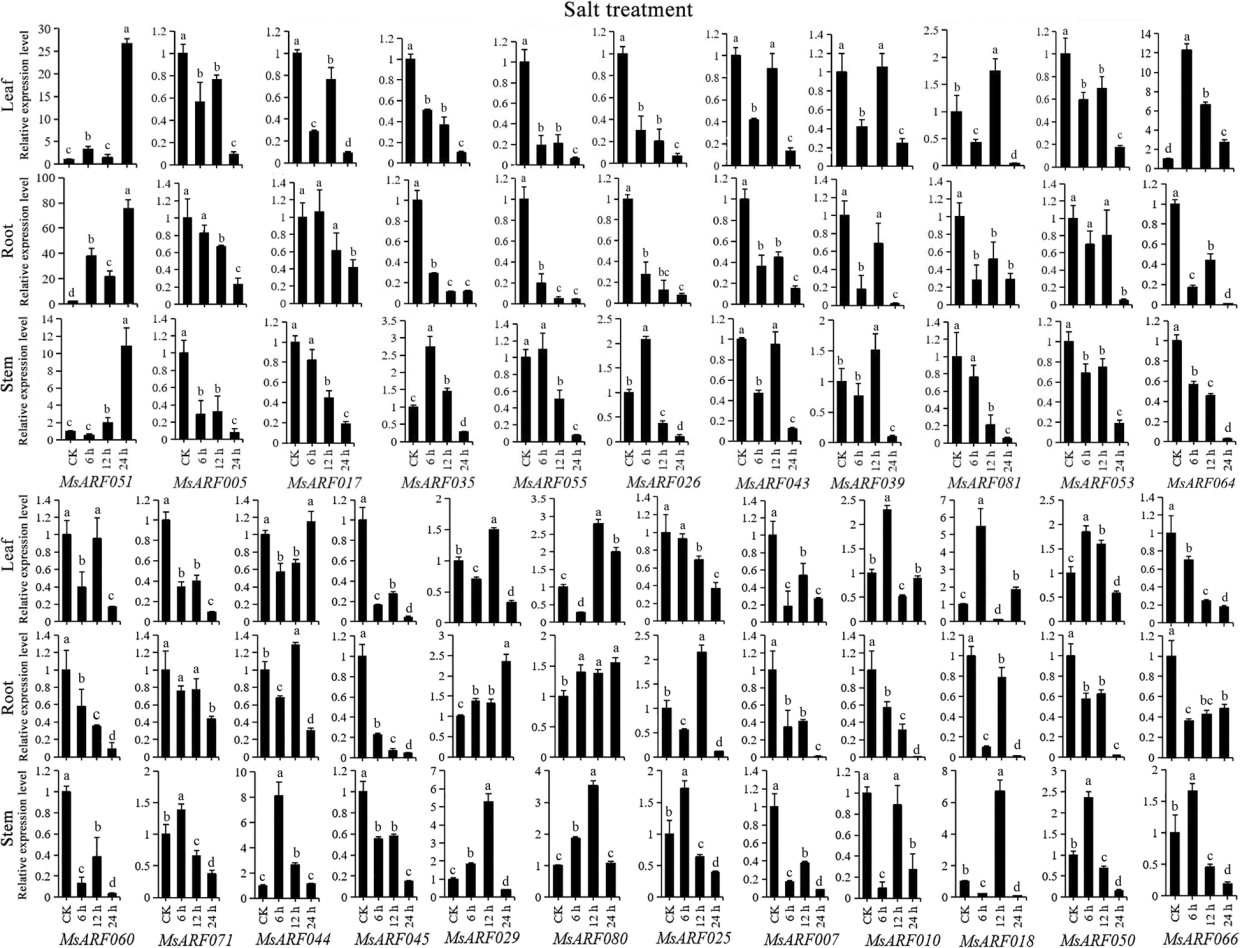
The expression pattern analysis of *ARF* genes in diverse organs undergoing salt stress treatment for 0, 6, 12, and 24 h using qRT-PCR. Data are presented as the mean ± standard deviation. Lowercase letters indicate a significant difference at the 5% level. The same below

**Fig. 9:**
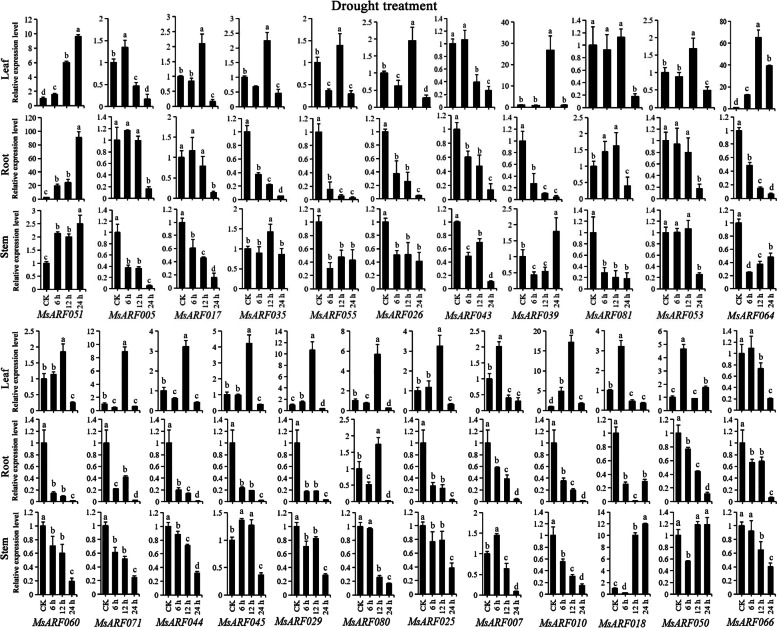
The expression pattern analysis of *ARF* genes in diverse organs undergoing drought stress treatment for 0, 6, 12, and 24 h using qRT-PCR

To verify the function of *MsARF*, a total of 25 *MsARF* genes (*MsARF005*, *MsARF007*, *MsARF010*, *MsARF017*, *MsARF018*, *MsARF025*, *MsARF026*, *MsARF029*, *MsARF035*, *MsARF039*, *MsARF041*, *MsARF043*, *MsARF044*, *MsARF045*, *MsARF047*, *MsARF050*, *MsARF051*, *MsARF053*, *MsARF055*, *MsARF060*, *MsARF064*, *MsARF066*, *MsARF071*, *MsARF080*, *MsARF081*) were selected to have their expression patterns analyzed during salt, drought, high-temperature, and low-temperature stresses at different time points (Table S8), based on RNA-seq data (Fig. 7B). *MsARF041* and *MsARF047* showed no or very low expression and were not further analyzed. The expression pattern of *MsARF*s under salt stress is depicted in Fig. 8. Except for *MsARF051*, which is upregulated under salt stress, other genes showed a downregulated trend compared to the control (CK). In leaf tissue, *MsARF051* at 24 h, *MsARF081* and *MsARF029* at 12 h, *ARF080* at 12 and 24 h, *ARF010* at 6 h, *MsARF018* at 6 and 24 h, and *MsARF050* at 6 and 12 h were all upregulated, other genes at other times were all downregulated. In root tissue, only *MsARF044* and *MsARF029* at 12 h, and *MsARF025* at 24 h were upregulated. In stem tissue, the expressions of *MsARF* genes were diverse. Figure 9 shows the expression patterns of 23 *MsARF* genes under drought stress. *MsARF051* was upregulated under drought stress in all three tissues compared to CK. The changes in other genes were relatively diverse and tissue-specific, as well as related to the duration of treatment. Addition to, most genes (*MsARF007*, *MsARF010*, *MsARF017*, *MsARF018*, *MsARF025*, *MsARF029*, *MsARF035*, *MsARF039*, *MsARF044*, *MsARF045*, *MsARF050*, *MsARF051*, *MsARF053*, *MsARF055*, *MsARF060*, *MsARF064*, *MsARF071* and *MsARF080*) were mainly upregulated in the leaves at 12 h.

**Fig. 10:**
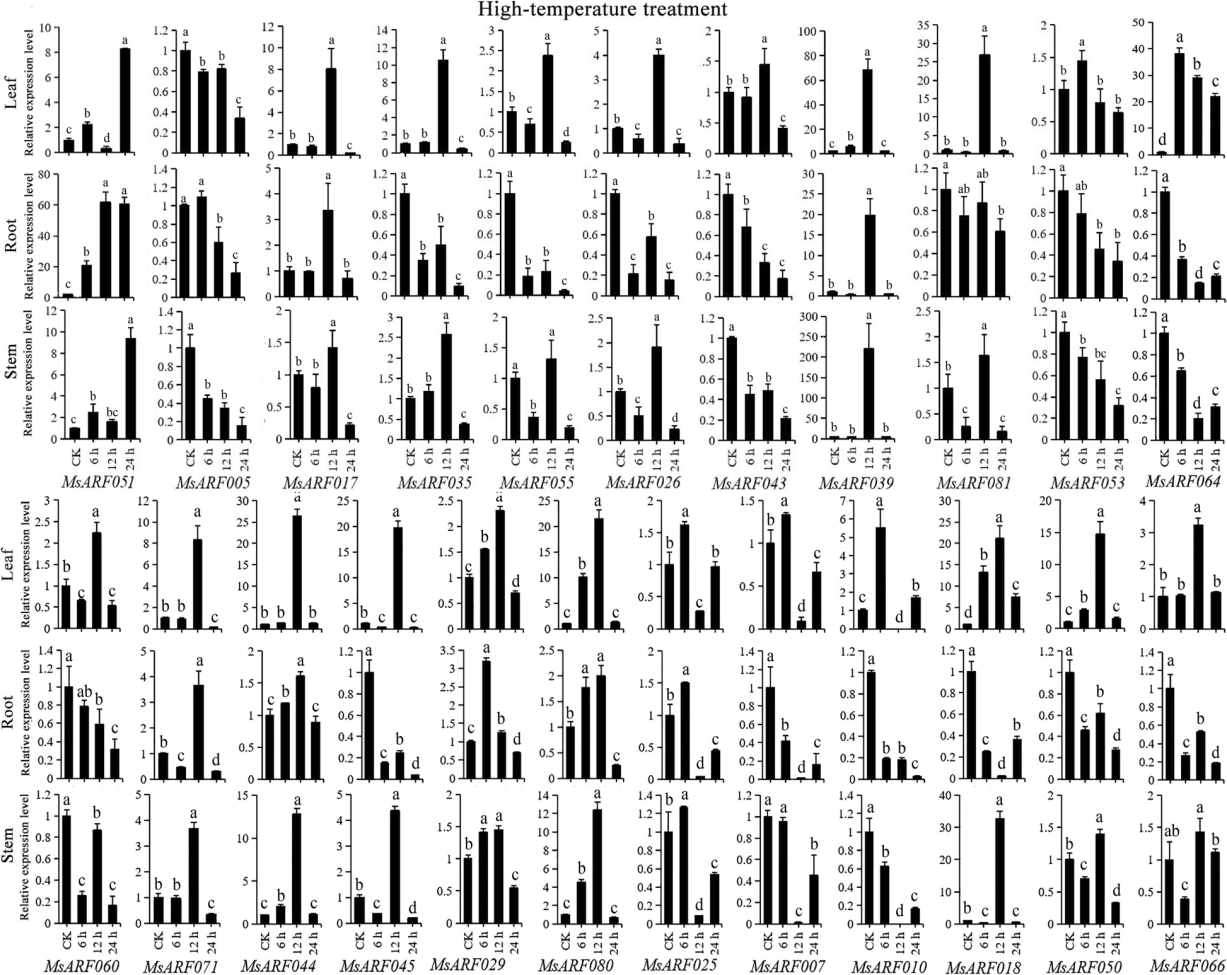
The expression pattern analysis of *ARF* genes in diverse organs undergoing high-temperature stress treatment for 0, 6, 12, and 24 h using qRT-PCR

**Fig. 11:**
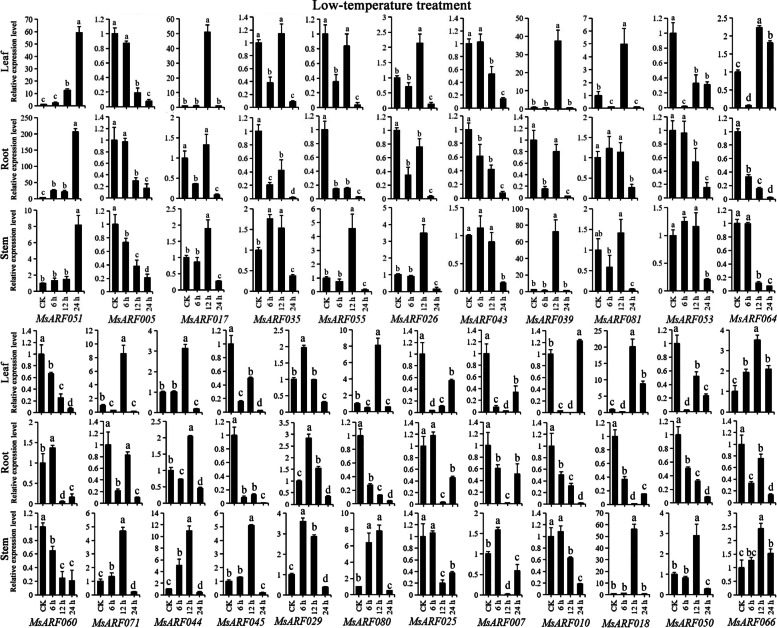
The expression pattern analysis of *ARF* genes in diverse organs undergoing low-temperature stress treatment for 0, 6, 12, and 24 h using qRT-PCR

Under high-temperature treatment, *MsARF051* was upregulated in all three tissues, and treatment time lengths compared with CK (Fig. 10). *MsARF017*, *MsARF039* and *MsARF071* were upregulated at 12 h, indicating that their heat stress regulation was tissue-independent and mainly acted at 12 h. Meanwhile, we also found that most *MsARF* genes were upregulated at 12 h in leaves and stems undergoing heat stress, while the expression pattern was quite varied in roots. Under low-temperature treatment, *MsARF051* was upregulated in all three tissues and treatments compared with CK (Fig. 11). *MsARF026*, *MsARF039*, *MsARF071* and *MsARF081* were upregulated at 12 h in leaf and stem tissues, with significantly distinct expression patterns in the roots. The changes in the remaining genes were tissue-specific and related to the treatment time length, And most genes were upregulated in specific tissues only at 12 h, suggesting that the regulation of cold stress by these genes was significantly correlated with the stress length. Compared with CK, *MsARF017*, *MsARF035*, *MsARF026*, and *MsARF043* were found in the leaves, and *MsARF017* was upregulated in the stem at 12 h and was downregulated at 6 and 24 h (Fig. S2). And compared to CK, *MsARF060* in roots and stems, while *MsARF044* in all three tissues were downregulated. The dynamic expression patterns of the remaining genes showed diversity.

## Discussion

Extreme weather conditions like drought, salt stress, and temperature have had a significant negative impact on alfalfa, one of the most economically important leguminous forages in the world. As a result, yield and geographic distribution have become major bottlenecks. As an important component of the auxin signaling pathway, *ARF* directly binds to and regulates the specific expression of downstream target genes during the auxin response [35]. Previous studies have shown that the *ARF* family plays a crucial role in plant growth, development, hormone response, and stress response [36, 37], making it a key gene family for understanding plant biology. Understanding the dynamics of this gene family provides an important resource for improving yield through variety improvement. However, there are no studies on the molecular function of alfalfa ARF transcription factors in response to abiotic stress. Therefore, studying the main structure and expression characteristics of *ARF* genes in alfalfa helps us gain insight into the regulatory role of *ARF* genes in alfalfa resistance to abiotic stress, growth, and development. This study identified 81 *ARF* family members in the alfalfa genome and analyzed the possible role of *MsARF* in different tissues and abiotic stress responses in alfalfa for further studies on the function of *ARF* genes.

### Members of the *ARF* family have revealed the evolution of tetraploid cultivated alfalfa

As a prominent effector of many aspects of the auxin response in plants, ARF transcription factors convert chemical signals into the transcriptional regulation of a group of specific genes [19]. Due to its functional importance in the auxin cascade, the *ARF* gene family has been deeply studied in model plants and major crops. The genome size of lettuce is 2.5 gigabases (Gb), which is considered one of the largest assembled plant genomes to date [38]. Compared to lettuce, the reference genome of autotetraploid alfalfa is larger, reaching up to 2.738 Gb [26]. Autotetraploid alfalfa can encode a larger *ARF* gene family based on such a large genome. This study identified 81 members of the *MsARF* family, which was greater than several reported crops, such as soybean (51), rice (25), and maize (36) [39–41]. Previous studies have shown that the genetic relationship between alfalfa and *Medicago truncatula* is closest [26]. A total of 40 *MtARF* genes were previously identified from the genome of diploid alfalfa [42], which is precisely half of the results of this study. These results may be related to the polyploidy of alfalfa (diploid genome), similar to the MADS-box genome-wide analysis of wheat [43]. Genome doubling (plant polyploidization) provides primitive genetic material for biological evolution, improves species diversity and environmental adaptability [44], and plays a vital role in the adaptive evolution of angiosperms [45]. Therefore, identifying the tetraploid cultivated alfalfa *ARF* family from the genome level provides essential theoretical guidance and practical application value for research that examines the biological evolution, species protection, and genetic breeding of subsequent alfalfa.

### Analysis of physicochemical properties of *MsARF* gene encoding proteins

This study’s identification and analysis of the *MsARF* gene family was completed using the whole genome data of the “Xinjiang Daye” alfalfa cultivar. The hydrophilicity analysis of 81 MsARF proteins showed that the instability coefficient of the MsARF protein family ranged from 22.84 (MsARF032) to 98.98 (MsARF039), but the instability index of most genes (66) was higher than 40, indicating that they were not stable proteins. The aliphatic amino acid index of the 81 MsARF proteins ranged from 48.43 (MsARF067) to 93.76 (MsARF001), all of which were less than 100, indicating that they were hydrophilic proteins. The further subdivision of the protein family was carried out using the protein hydrophobicity coefficient. Among them, 22 MsARF proteins had a hydrophobicity coefficient less than − 0.5, indicating that they were hydrophilic proteins, while the other 59 MsARF proteins were between − 0.5 and 0.5, indicating that they were amphiphilic proteins. This result indicates that the MsARF family proteins have better hydrophilicity. As transcription factors, ARF proteins usually function in the nucleus [7]. Consistent with the subcellular localization results reported in other species, such as eggplant [7], tomato [46], and maize [41], the subcellular predictions indicate that most of the *MsARF* genes were localized to the nucleus. In addition, no *MsARF* genes were found to have any conducting peptides, signal peptides, or transmembrane structures, indicating that the 50 *MsARF* genes located in the nucleus did not secrete or transport but only regulated gene expression in the nucleus.

### *MsARF *gene homology analysis

A phylogenetic tree was constructed to analyze the relationship between the *ARF* gene family in rice, *Arabidopsis*, and cultivated alfalfa (Fig. 2). The *ARF* gene family was divided into four large clades, where clade I contained the most *MsARF* (52) genes and was closely related to *AtARF10*/*16*/*17*. *AtARF10/16* controls root crown cell formation, limiting the stem cell niche and promoting columella cell differentiation [47]. *AtARF10* plays an important role in regulating the formation of the primary outer wall and pollen development in *Arabidopsis* [48]. Therefore, we hypothesized that these 52 *MsARF* genes have similar functions to *AtARF10/16/17*. Clade II contained 13 alfalfa genes that were closely related to *AtARF5/6/7/8/19*, and it has been speculated that these 13 *MsARF* genes may be auxin-promoting factors in carrot protoplasts [49]. Clade III contained seven alfalfa genes, which were closely related to *AtARF3* and *AtARF4*, and it has been speculated that these seven *MsARF* may be auxin suppressors [49]. Clade IV contained nine alfalfa genes, which were closely related to 13 *Arabidopsis* genes such as *AtARF1*, *AtARF2*, and *AtARF11*, suggesting that these nine *MsARF* may act as auxin repressors involved in the Abscisic Acid (ABA) pathway regulating seed germination and primary root growth [50].

### The analysis of the *MsARF* gene structure

Based on the phylogenetic tree, analysis of ten conserved motifs of *MsARF* genes revealed different clades of *MsARF* genes containing common or specific motifs (Fig. 3). Although the number of motif members varied among clades, the group motif patterns were strongly conserved. The comparison of novel functional domains or motif sequences across multiple homologous proteins has been a widely used method to predict protein function based on evolutionary conservation [7]. We found that motif 8 was highly conserved in the *MsARF* gene family. In addition, we also found that motif 7 was highly conserved in the *MsARF* subfamily in Clades II, III, and IV. Although most of the motifs in *MsARF* were conserved, other motifs may have been related to novel plant functions and should be studied further. By analyzing the conserved domains of MsARF protein sequences, we found that all *MsARFs* contain an auxin resp domain, and 71 *MsARFs* contain a typical DBD domain (B3-like), while only 19 (23.46%) *MsARFs* contain an auxin/IAA binding domain and are mainly distributed in clades II, III, and IV. This result was similar to the conserved domain of the ARF protein sequence in *Medicago truncatula*, indicating that auxin can also regulate *MsARF* genes independently [51].

To further determine the structural differences between *MsARF* genes, gene structure analysis was conducted on the *MsARF* family. The number of CDS in the *MsARF* gene ranged from 1 to 15, and no UTR region was identified. This finding was consistent with the previous reports in papaya [9], maize [41], and barley [52], indicating that the gene structure of the *ARF* family was relatively simple. Gene structure analysis determined the structural differences among the *MsARF* gene family. Analysis of the distribution of 7 *cis*-regulatory elements in the 2000 bp (bp) upstream promoter region of the *MsARF* revealed that ABRE had the most *cis*-regulatory elements, followed by CGTCA motifs, MBS, TC-rich repeats, TGA elements, LTR, and AuxRR cores (Fig. 5), indicating that *MsARF* played a positive role in hormone responses to plant stressors.

### Chromosomal localization and collinearity analysis of the *MsARF*

We found that 81 *MsARF* genes were unevenly distributed on 25 chromosomes (Chr) of alfalfa and were the most widely distributed on chromosome 5, indicating that *MsARF* genes mainly perform biological functions on chromosome 5. Gene replication is traditionally considered a way to expand and obtain functional diversity during evolution [53]. Gene duplication analysis in the *MsARF* gene family showed that fragment duplication had a greater impact on *ARF* genes, which was consistent with the results observed in rice and *Arabidopsi*s in previous studies [54], suggesting that replication events in the *ARF* family within a plant genome may be a general evolutionary mechanism. Ka/Ks ratios are usually an important indicator of selection pressure in evolution [34]. The analysis of homologous *MsARF* gene pairs showed that only 2 pairs of fragment replicates (*MsARF008/MsMASS042* and *MsARF008/MsMASS042*) had a Ka/Ks ratio greater than 1, while the remaining 14 pairs of *MsARF* had a Ka/Ks ratio less than 1, indicating that they experienced strong purification selection after duplication.

### The *MsARF* genes function in response to abiotic stress

Since *ARF* genes play a vital role in controlling phytohormone signaling, studying each family member’s function is necessary. ARF transcription factors are crucial in plant growth and development and actively participate in plant resistance to stress [6, 17, 19, 20]. This study combined transcriptome data with a novel bioinformatic approach to search for *MsARF* genes that have functions related to the response and regulation of drought stress in alfalfa. We compared the *MsARF* genes with known transcriptome data and initially selected genes that were significantly differentially expressed under drought, salt, and cold stress as *ARF* candidate genes responding to abiotic stress. Subsequently, alfalfa seedlings were treated with salt, drought, cold, and heat stress, and qRT-PCR experimentally verified their expression patterns. As reported by Zhou et al. [55], *ARF12* might play central roles in the regulation of NaCl-responsive genes. In addition, *AcARF5* can positively regulate salt stress and drought stress in kiwifruit (*Actinidia chinensis*) [56]. When compared to CK, *MsARF051* was upregulated in all treatment groups, which helped us elucidate that *MsARF051* had great potential for transformation in the cultivation of reverse-resistant alfalfa varieties. In addition, *MsARF017* and *MsARF039*, *MsARF026, MsARF071* and *MsARF081* were upregulated during temperature stress, and *MsARF039* could regulate heat stress and cold stress in alfalfa. Furthermore, we found that most of the *ARF* genes were upregulated at 12 h under all abiotic stresses involved in this study, and we speculated that these *ARF* genes were mainly active at 12 h and belong to short-acting genes, especially in the leaves and stems. Future studies focusing on alfalfa should examine these genes and conduct further research through genetic engineering and editing techniques in our research. The results of this study on the alfalfa *ARF* gene family at the whole genome level create a foundation for mining stress resistance genes for alfalfa and provide an in-depth analysis of abiotic stress theory and guidance for the creation of new germplasm using genetic engineering technology for an alfalfa variety that has increased stress resistance.

## Conclusion

Based on the whole genome of cultivated alfalfa, this study identified the *ARF* gene family, predicted the basic physical and chemical properties of the genes, and analyzed the phylogenetic relationship, gene structures, chromosomal localization, paralogous and homologous genes, and expression patterns of *ARF* genes in response to abiotic stress and different tissues. A total of 81 *MsARF* genes were identified and phylogenetically divided into four branches, analyzing 14 discovered segmental repeat pairs and two sets of tandem repeats. Compared with the transcriptomic data of alfalfa, the expression of *MsARF051* under all stress treatments can be used as the candidate gene of alfalfa. *MsARF039* is mainly involved in the response of alfalfa to temperature stress and can be used to improve new alfalfa varieties cultivated in areas with extreme temperatures. Future research will examine the functional verification of these genes and analyze the genetic transformation of these two genes to lay a foundation for further investigation of the abiotic stress theory and the creation of new germplasm using genetic engineering technology for an alfalfa variety that has increased stress resistance.

## Materials and methods

### Materials

The material used in this experiment was cultivated “Xinjiang Daye” alfalfa, and its seeds were provided by the National Livestock Husbandry Station, Ministry of Agriculture and Rural Affairs of The People’s Republic of China.

### Identification of the *MsARF* gene family and analysis of its physical and chemical properties

The Plant Transcription Factor Database (http://planttfdb.gao-lab.org/) was used to download the *ARF* protein sequences from Arabidopsis and rice model plants. The genome data and protein-nucleic acid sequence of “Xinjiang DaYe” alfalfa used in this experiment were downloaded from the alfalfa genome website (https://figshare.com/projects/whole_genome_sequencing_and_assembly_of_Medicago_sativa/66380) [26]. In addition, the hidden Markov model (HMM) profile of ARF (PF06507) was downloaded from the Pfam database (http://pfam.xfam.org/), and MsARF family proteins from alfalfa genome were identified using HMMER 3.0 with an E-value set to 1.0 as the threshold and the remaining parameters set to default. The redundant protein was removed using the Expasy [57] online database (https://web.expasy.org/decrease_redundancy), with all parameters set to default values. Further characterization of the ARF domain in the MsARF protein sequences was performed using the Pfam website and NCBI-CD Search (https://www.ncbi.nlm.nih.gov/Structure/cdd/wrpsb.Cgi) to verify the accuracy of the MsARF protein sequences after the removal of the redundancy. Sequences without the ARF domain were removed [58]. A total of 81 MsARF protein sequences were subsequently analyzed for physicochemical properties using the online network ProtParam (https://web.expasy.org/protparam/) tools, including molecular weight (MW), theoretical isoelectric point (pI), instability index, and the grand average of hydropathicity (GRAVY) index. Subcellular localization (https://www.genscript.com/wolf psort.html) was predicted for all 81 *MsARF* genes by the online tool WoLF PSORT. The predicted genes were named *MsARF001* through *MsARF08*1 according to their order of occurrence in the genome. In addition, GO and KEGG annotations were performed using the Alfalfa Database (http://47.92.172.28:12088/) to investigate the functional role of the *MsARF* genes [59, 60].

### Phylogenetic analysis, gene structure and motif composition of the *MsARF*s

To analyze the evolutionary relationship between MsARF proteins, multiple sequence alignments of MsARF, AtARF, and OsARF protein sequences were performed using MEGA 7.0 software [61] and automatically trimmed with TrimAL [62]. The maximum likelihood phylogenetic tree was constructed from IQ-TREE version 1.6.12, and the JTT + R6 model was determined to be the best sub-phylogenetic model using ModelFinder, with the Ultrafast bootstrap set to 1000 [63, 64]. Based on the evolutionary relationship between *MsARF* genes and *AtARF*, *MsARF* genes were classified into a clade I-IV type [65]. The MEME 4.12.0 online tool (http://meme-suite.org/) was used to identify the conserved motifs of the 81 alfalfa MsARF proteins. The parameters were set as follows: The maximum number of motifs was 10; each sequence selected a zero or one occurrence as the site distribution; the minimum and maximum motif widths were set to 6 and 200, respectively; other parameters were set to default values; and the motif features were visualized with TBtools [33].

### Gene duplication and gene structure analysis

CDS sequences and gene sequences corresponding to all *MsARF* genes were obtained from the alfalfa genome file for prediction analysis of intron and exon structures of *MsARF* genes using GSDS2.0 (gene structure display server) (http://gsds.gao-lab.org/). MapGene2Chrome V2.0 (http://mg2c.iask.in/mg2c_v2.0/) was used to map chromosome localization based on the alfalfa genome annotation file (GFF3) data to explore the distribution characteristics of *ARF* genes on chromosomes. Based on the alfalfa genome sequence file in fasta format and the corresponding gene structure annotation file in GFF3/GTF format, referring to the method of Chen et al. [33], TBtools software was used to complete the chromosome location of genes and the collinearity analysis of gene duplications. The specific steps: first, the “Fasta Stats” function was opened, the alfalfa genome file was input, and the chromosome length information file was obtained. Next, the “Gene Density Profile” function was used, and gene structure annotation files was input to obtain gene density information. Then, by using the “one step MCScanX-super fast” function, the alfalfa genome file and corresponding gene structure annotation file were input to obtain the collinearity file of genes within the species. Subsequently, the association file between genes was obtained through the “File Merge for MCScanX” and “File Transform for MicroSynteny Viewer” functions, and the green RGB value was added to the *MsARF* gene pairs, as well as the red RGB value was added to the tandem repeat sequence genes. Finally, the “Advanced Circos” function was used to input the file obtained in the previous steps to draw the circos diagram.

### Protein signal peptide, guide peptide, transmembrane structure and protein secondary and tertiary structure analysis

The signal peptide and transmembrane structures of the *MsARF* were analyzed using SignalP 5.0 (https://services.healthtech.dtu.dk/services/SignalP-5.0/) and TMHMM 2.0 (https://services.healthtech.dtu.dk/services/TMHMM-2.0/), respectively. The conducting, or guide, peptide analysis of the *MsARF* was also performed using the Target P-2.0 Server (https://services.healthtech.dtu.dk/services/TargetP-2.0/). The MsARF protein secondary structure was analyzed using SOPMA (https://npsa-prabi.ibcp.fr/cgi-bin/npsa_automat.plpage=npsa_sopma.html) to obtain the percentage of different conformations, including α -helix, extended chain, β -turn angle, and random coil. Using SWISS-MODEL (http://swissmodel.expasy.org/interactive), this structural analysis was subsequently used to analyze the tertiary structure of MsARF proteins.

### ***Cis***-regulatory element analysis

The PlantCARE database (http://bioinformatics.psb.ugent.be/webtools/plantcare/html/) analyzed *cis*-regulatory elements in the 2000-bp sequence upstream of the upstream transcription start site of the 81 *MsARF* genes. In the promoter region of alfalfa *ARF* genes, seven *cis*-regulatory elements known to associate with stress and hormones were identified, including ABRE, TC-rich repeats, MBS, LTR, CGTCA-motif, AuxRR-core, and TGA-element, which are involved in the abscisic acid response, defense and stress response, drought induction, low-temperature response, Methyl Jasmonate (MeJA) response, auxin response, and auxin response *cis*-elements.

### *MsARF *gene in response to abiotic stress

To investigate the expression level of *MsARF* under abiotic stress and various tissue sites, BLASTn alignment with the Alfalfa Database produced the expression profile data of *MsARF* genes under abiotic stress and various tissue sites. To deeply investigate the expression patterns of *MsARF* genes in different tissues, expression profile data from eight tissues were analyzed, including flowers, leaves, roots, post-elongating stems, nodules, elongating stems, young leaves, mature leaves, and senescent leaf tissues of alfalfa. The expression analysis of *MsARF* genes under abiotic stress included the transcriptional expression profiles of alfalfa under cold, drought, salt, aluminum, and lead stress. OmicShare Tools (https://www.omicshare.com/tools/), a freely available data analysis platform, was used for cluster analysis and expression quantity heat map mapping, and the expression level was expressed by Z-core normalized calculation results for the expression data.

### Growth and stress treatment of plant materials

The plant material used in this experiment was “Xinjiang DaYe” alfalfa. First, we chose seeds with healthy and identical shapes. After surface sterilization of alfalfa seeds, they were planted in pots with vermiculite: nutrient soil = 1:1 [20 centimeters (cm) diameter, 20 cm high] with 5 biological replicates per treatment group. The seedlings were cultured in a 16-hour light/8-hour dark cycle with 60% relative humidity and a temperature of 22 °C. After 15 days, the alfalfa seedlings were inoculated with different stress treatments. A 15% polyethylene glycol-6000 solution was added to the seedling pots by root irrigation (until thoroughly poured) to simulate drought. To simulate salt stress, 220 micromolar (mM) sodium chloride solution was added to the seedling pots by root irrigation (until thoroughly poured). The whole alfalfa seedlings were harvested at different stress time points (6, 12, and 24 h) for the drought treatment group and the salt stress treatment group. To simulate cold stress, alfalfa seedlings to be treated were transferred to a 16-hour light/8-hour dark cycle in an artificial climate chamber with 60% relative humidity and temperatures at 5 °C. To simulate high temperature stress, alfalfa seedlings to be treated were transferred to a 16-hour light (32 °C)/8-hour dark (28 °C) cycle in an artificial climate chamber with 60% relative humidity. Whole alfalfa seedlings were collected after 6, 12, and 24 h of incubation. All samples were flash-frozen in liquid nitrogen and stored at 80 °C for downstream analysis.

### RNA extraction and qRT-PCR

To verify the response pattern of *MsARF* genes to abiotic stress, twelve genes were selected for qRT-PCR analysis based on the expression heatmap. According to the instructions of the manufacturer, total ribonucleic acid (RNA)from the root, stem, and leaf tissues of “Xinjiang Daye” alfalfa under different stress treatment times were extracted using an RNA Simple Total RNA kit (Tiangen, Shanghai, China) and were reverse transcribed into cDNA using a FastKing cDNA kit (Tiangen, Shanghai, China). Using the Primer-BLAST tool at NCBI (https://www.ncbi.nlm.nih.gov/tools/primer-blast/), twelve gene-specific PCR primers were designed (Table S9) for gene expression analysis. The QuantStudio 5 real-time PCR system (Thermo Scientific, Massachusetts, USA) was used to amplify samples and standards with Hyperreal Premix Plus (SYBR Green) (Tiangen, Shanghai, China). Three biological replicates were used. Chen et al. described the experimental process that we used as a model for our experimentation [61]. The relative transcription levels of the selected genes were calculated with the 2^−ΔΔCT^ method [62] and normalized to the expression levels of the *Medicago truncatula ACTIN* gene (AES78237. 1). The qRT-PCR data were collated and plotted in Excel 2016, and the one-way ANOVA (*p* < 0.05) was analyzed by IBM SPSS 24.0 software.

### Supplementary Information

**Additional file 1: Table S1.** List of the ARF sequences in Alfalfa. **Table S2.** Protein property of ARF proteins. **Table S3.** Gene ontology (GO) annotation results of ARF genes. **Table S4.** Kyoto Encyclopedia of Genes and Genomes (KEGG) annotation results of ARF genes. **Table S5.** The subcellular localization and secondary structure analysis of ARF proteins. **Table S6.** Ka and Ks of MsARF gene pairs. **Table S7.** Expression (FPKM) of ARF genes in various alfalfa tissues in response to cold, drought, salt, aluminum, and lead treatment. **Table S8.** Relative expression data of 23 MsARF genes related to abiotic stress. **Table S9.** Sequences of ARF primers used in qRT-PCR.

**Additional file 2: Fig. S1.** Conserved Motif of ARF proteins. **Fig. S2.** Relative expression level analysis of ten *ARF* genes without stress treatment for 0, 6, 12, and 24 hours using qRT-PCR.

## Data Availability

All data generated or analyzed in this study are included in this published article and its supplementary material. The draft genome data of autotetraploid cultivated (‘Xinjiang Daye’) alfalfa was obtained from figshare (https://figshare.com/projects/whole_genome_sequencing_and_assembly_of_Medicago_sativa/66380). The *Arabidopsis* and rice ARF protein sequences were all downloaded from Plant Transcription Factor Database (http://planttfdb.gao-lab.org/). Genome-wide transcriptome data of different alfalfa tissues were acquired from the *Medicago* Analysis Portal (https://medicago.legumeinfo.org/). All transcriptome sequencing data analysed during the current study are available in the NCBI SRA repository (https://www.ncbi.nlm.nih.gov/sra/): SRR7091780-SRR7091794 (cold treatment), SRR7160322-SRR7160357 (drought and salt treatments), SRR22519684-SRR22519695 (aluminum stress), and SRR5279707-SRR5279711 (lead stress).

## Abbreviations

ARF: Auxin-Response Factor
GO: Gene Ontology
KEGG: Kyoto Encyclopedia of Genes and Genomes
qRT-PCR: Quantitative reverse transcription polymerase chain reaction
DBD: DNA binding domain
AuxRE: Auxin response elements
MR: Variable intermediate region
AD: Activating domain
RD: Inhibitory domain
CTD: C-terminal dimerization domain
Chr: Chromosome
Ka/Ks: Nonsynonymous substitution rate/synonymous substitution rate
FPKM: Fragments Per Kilobase of transcript per Million mapped reads
