# Supplementary material for: Genome-wide identification and expression analysis of the Auxin-Response factor (ARF) gene family in Medicago sativa under abiotic stress
Source: BMC Genomics. 2023 Aug 29;24:498. doi: 10.1186/s12864-023-09610-z (PMC10463752; doi:10.1186/s12864-023-09610-z)
Supplement: Supplementary file 2 — Additional file 2: Fig. S1. Conserved Motif of ARF proteins. Fig. S2. Relative expression level analysis of ten ARF genes without stress treatment for 0, 6, 12, and 24 hours using qRT-PCR. [file 12864_2023_9610_MOESM2_ESM.docx]

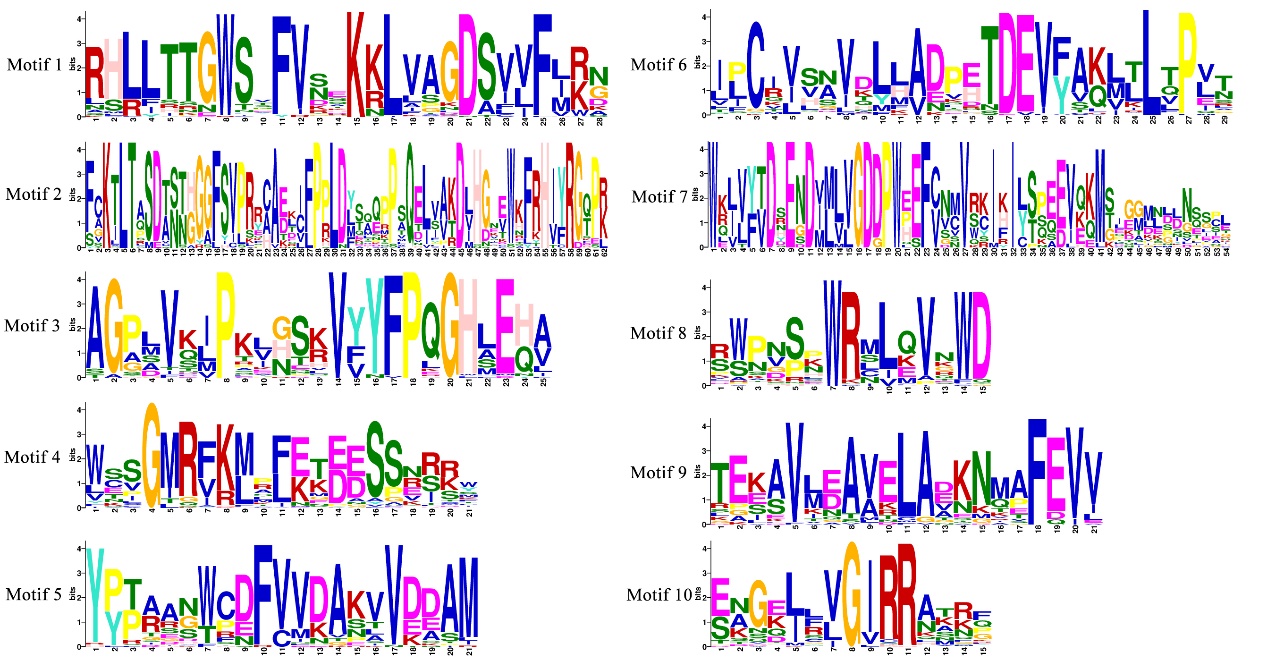


Fig. S1 Conserved Motif of ARF proteins.


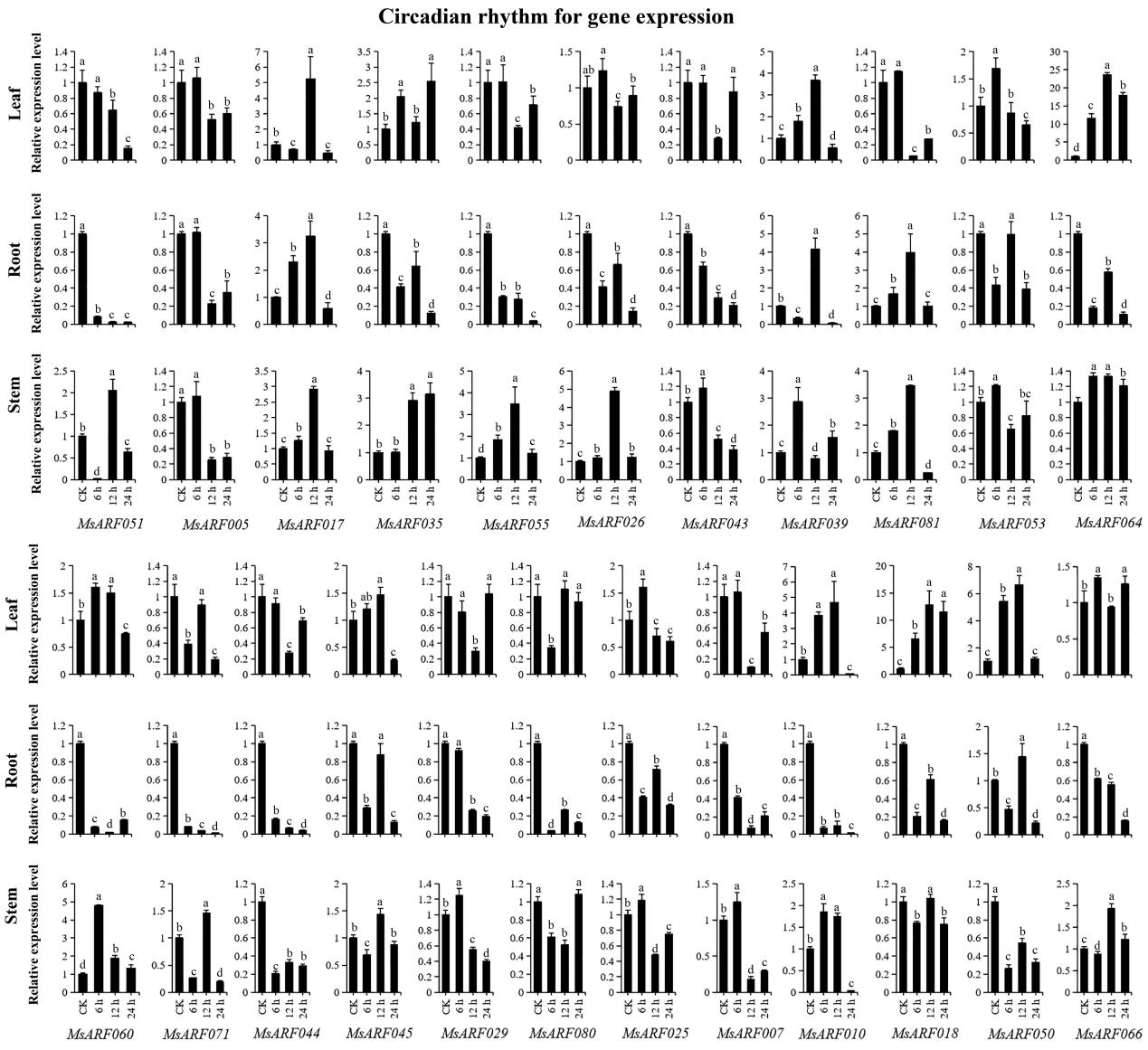


Fig. S2 Relative expression level analysis of ten *ARF* genes without stress treatment for 0, 6, 12, and 24 hours using qRT-PCR.
